# The utility of anti-Müllerian hormone in the diagnosis and prediction of loss of ovarian function following chemotherapy for early breast cancer

**DOI:** 10.1016/j.ejca.2017.10.001

**Published:** 2017-12

**Authors:** R.A. Anderson, J. Mansi, R.E. Coleman, D.J.A. Adamson, R.C.F. Leonard

**Affiliations:** a MRC Centre for Reproductive Health, University of Edinburgh, Edinburgh, UK; b Department of Oncology, Guy's and St Thomas' NHS Foundation Trust and Biomedical Research Centre, King's College London, UK; c Department of Oncology, Sheffield University, Sheffield, UK; d Tayside Cancer Centre, Ward 32, Ninewells Hospital Dundee, UK; e Department of Surgery and Oncology, Imperial College London, UK

**Keywords:** Breast cancer, Hormone sensitive, Menopause, Ovarian function

## Abstract

**Aim:**

Chemotherapy results in permanent loss of ovarian function in some premenopausal women. Accurate identification in women with hormone-sensitive early breast cancer (eBC) would allow optimisation of subsequent endocrine treatment. We sought to assess whether analysis of anti-Müllerian hormone (AMH) using a sensitive automated assay could identify women who would not regain ovarian function after chemotherapy.

**Methods:**

Data from women in the Ovarian Protection Trial in Premenopausal Breast Cancer Patients (OPTION) trial of goserelin (a gonadotrophin-releasing hormone (GnRH) analogue) for ovarian protection were analysed. Women were assessed for premature ovarian insufficiency (POI: amenorrhoea with elevated follicle-stimulating hormone (FSH)) at 24 months after diagnosis. The accuracy of AMH for the diagnosis of POI and its prediction from measurement at the end of chemotherapy was calculated.

**Results:**

AMH below the level of detection showed good diagnostic accuracy for POI at 24 months (n = 73) with receiver operating characteristic (ROC) area under the curve of 0.86, sensitivity 1.0 and specificity 0.73 at the assay limit of detection. In women aged >40 at diagnosis who did not receive goserelin, AMH measured at end of chemotherapy also gave good prediction of POI at 24 months (area under the curve (AUC) 0.89 95% CI 0.75–1.0, n = 32), with sensitivity 0.91, specificity 0.82, diagnostic odds ratio (DOR) 42.8. FSH gave slightly lower AUC, and specificity was low at 0.55. Age but not tamoxifen impacted on AMH levels.

**Conclusion:**

Using this sensitive AMH assay, the finding of an undetectable AMH level in women aged >40 at the end of chemotherapy for eBC gave a good prediction that ovarian function would not return. This may allow alterations in post-chemotherapy endocrine management.

## Highlights

• Chemotherapy for early breast cancer results in permanent loss of ovarian function in some women.
• We assessed the accuracy of measurement of anti-Müllerian hormone (AMH) at end of chemotherapy for prediction of this in women aged >40 at diagnosis.
• AMH measured at end of chemotherapy gave good prediction of premature ovarian insufficiency (POI) at 24 months.
• This may allow optimisation of subsequent endocrine treatment.

## 1 Introduction

Suppression of ovarian function or blockade of oestrogen production or synthesis is a key part of the treatment of hormone receptor-positive breast cancer [1]. In premenopausal women suppression of oestrogen production can be achieved by concurrent administration of a gonadotrophin-releasing hormone (GnRH) analogue [2]. The recent trials (Suppression of Ovarian Function Trial [SOFT] and Tamoxifen and Exemestane Trial [TEXT]) confirmed the benefit of endocrine therapy to suppress ovarian function in reducing recurrence rate, although not overall survival [3], [4]. However, this has adverse consequences for patient's quality of life [5].

Although the loss of growing ovarian follicles during chemotherapy frequently results in women developing amenorrhoea [6], [7], many subsequently regain ovarian function and thus, chemotherapy-induced amenorrhoea does not reliably demonstrate postmenopausal status [8]. The likelihood of ovarian recovery depends on the chemotherapeutic regimen, the patient's age, and pre-existing ovarian reserve [6], [9], [10], [11], [12], [13] but there are at present no diagnostic tests or predictors of recovery of sufficient accuracy for clinical use. A more accurate assessment of ovarian function post chemotherapy would be valuable and might aid selection of better endocrine therapy after chemotherapy.

Anti-Müllerian hormone (AMH) is produced by small growing follicles [14]. Their number indirectly reflects the number of remaining primordial follicles, the true ovarian reserve, necessary for ongoing ovarian function. Serum AMH falls rapidly during chemotherapy [15], [16], with variable recovery thereafter reflecting the degree of ovarian damage and thus post-treatment ovarian function [9], [17], [18], [19]. AMH assays have previously been insufficiently sensitive to be of great value in diagnosing the menopause, becoming undetectable several years prior to final menses [20], but recent technological developments have resulted in markedly improved assay sensitivity [21]. Using one such highly sensitive assay, we have shown that women who were premenopausal at breast cancer diagnosis but who subsequently develop amenorrhoea and undetectable AMH following chemotherapy are very likely to remain amenorrhoeic [22]. We report an analysis of serum AMH in relation to post-chemotherapy ovarian function in women treated for breast cancer as part of the OPTION trial, to assess the diagnostic accuracy of AMH for POI following recovery from chemotherapy, and the potential for early post-chemotherapy AMH levels to predict that recovery.

## 2 Methods

OPTION was a Randomised Controlled Trial (RCT) of the effect of goserelin administration during chemotherapy to reduce ovarian toxicity [23], Trial registration: EudraCT 2004-000133-11. In brief, the study population consisted of premenopausal women with histologically confirmed breast cancer who were to receive adjuvant or neo-adjuvant chemotherapy. Patients were randomised to receive goserelin 3.6 mg monthly from shortly before chemotherapy until the end of chemotherapy; regimens included 6–8 cycles of cyclophosphamide and/or anthracycline-containing regimens with or without a taxane. The primary outcome was the prevalence of amenorrhoea at 12–24 months after diagnosis, supported by hormone measurements to allow the diagnosis of premature ovarian insufficiency (POI), defined as amenorrhoea plus follicle-stimulating hormone (FSH) concentration >25 IU/l, with patients divided in two age cohorts, ≤40 versus >40 years at diagnosis. All patients gave informed consent, and the study received Ethical Committee approval.

Hormone analyses were available on a subset of women, with samples for this analysis taken pre-treatment, at the end of chemotherapy, and 12 and 24 months after diagnosis. FSH, oestradiol (E2) and AMH were measured in serum using the Roche Elecsys^®^ system. The AMH assay has a limit of detection of 0.07 pmol/l (0.010 ng/ml), the oestradiol assay (Oestradiol III) has a limit of detection of 18.4 pmol/l and limit of quantification of 61.3 pmol/l.

Hormone data were not normally distributed and are presented as median ±95% confidence intervals. Statistical analysis to assess hormone concentration changes from the end of chemotherapy was by Kruskal–-Wallis - test with Dunn's multiple comparison tests, with further analysis by menstrual function/POI, and age (≤40 versus >40 years at diagnosis). Analysis of diagnostic value was performed by generating ROC curves, and calculation of sensitivity, specificity and likelihood ratio (LR) at cut-off values of 0.07 pmol/l for AMH (the limit of detection) and 25 IU/l for FSH [24]. Cut-off values for AMH and FSH in analysis of pre-chemotherapy samples were derived from the combination of sensitivity and specificity giving the highest LR. Positive and negative predictive values (PPV and NPV), and diagnostic odd ratio (DOR) were also calculated. Data from all available patients were used for the diagnostic analysis in relation to whether or not women had POI (amenorrhoea between 12 and 24 months after diagnosis, with FSH>25 IU/l). The predictive analysis of hormonal data at end of chemotherapy versus later amenorrhoea/POI was confined to the control group who did not receive goserelin, to avoid any impact of goserelin on AMH and FSH levels [15]. Prediction of later POI by pre-treatment hormone concentrations was performed using data from all women as those time points were distant from goserelin administration. Baseline characteristics of women included in this analysis are given in Table 1.

**Table 1:** Baseline characteristics of women in these analyses.

	All women	Controls only
n	101	68
Age (years)	39.5 ± 0.5	39.5 ± 0.6
Proportion of controls	52%	100
Baseline FSH (IU/l)	8.1 ± 0.7	9.0 ± 0.9
Baseline AMH (pmol/l)	9.6 ± 1.2	8.2 ± 1.5

All data are mean ± sem. Data for the Control group are those women not treated with GnRHa who were included in the analysis of end of treatment AMH and FSH versus POI.

## 3 Results

**Fig. 1:**
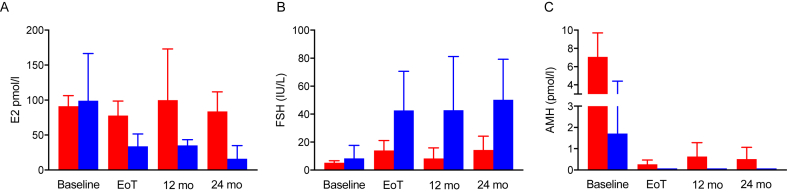
Serum oestradiol (A), FSH (B) and AMH (C) at pre-treatment baseline, end of treatment (EOT) and at 12 and 24 months after diagnosis, by POI at 24 months. Data from all women from the OPTION trial. Red, women without POI; blue: women with POI (amenorrhoea plus FSH >25IL/L at 24 months). N = 96 and 28 respectively; median ±95% confidence intervals.

Serum concentrations of E2, FSH and AMH during the course of the study are shown in Fig. 1. Compared with women with ovarian function at 24 months, E2 concentrations at the end of treatment were lower in women with subsequent POI (p = 0.0003) and continued to fall (p = 0.009 at 24 months versus end of treatment), with no changes beyond the end of treatment in women who did not develop POI. FSH was significantly higher at the end of treatment in women with POI (p = 0.001) and did not change thereafter, whereas there was a small fall after treatment completion in women without POI (p = 0.03). Women who developed POI had lower pre-treatment AMH concentrations (p = 0.0002) than those who did not. AMH concentrations were markedly reduced at the end of treatment in all women compared to pre-treatment, and lower in women who subsequently had POI than those who did not (p < 0.0001). From the end of treatment, AMH concentrations showed a small increase in women without POI at 12 months (p = 0.006) with no further rise at 24 months, and women with POI had lower AMH concentrations at 24 months than those who did not (p < 0.0001).

**Fig. 2:**
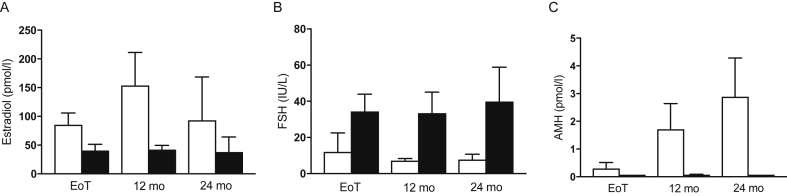
Serum oestradiol (A), FSH (B) and AMH (C) at end of treatment (EOT) and at 12 and 24 months after diagnosis in women aged ≤40 (open bars) versus >40 years (filled bars); n = 62 and 81, median ± 95% confidence intervals.

Younger women (≤40 years) showed a significant increase in AMH (p < 0.0001) and fall in FSH (p = 0.004) from the end of chemotherapy, whereas women aged over 40 years showed no significant post-treatment changes in these hormones (Fig. 2). AMH was detectable at the end of chemotherapy in 35% of women aged over 40, versus in 84% in women aged ≤40 (p = 0.0002). There were no changes in E2 in either age group although it tended to be higher and more variable in the younger group. Tamoxifen was taken by 38% of women, equally distributed by POI (p = 0.6). AMH concentrations at both 12 and 24 months were unaffected by tamoxifen administration (at 24 months: 2.5 ± 0.9 with tamoxifen versus 2.1 ± 0.7 pmol/l) and the distinction by POI was unchanged (with tamoxifen: AMH in POI 0.07 ± 0.0 versus not POI 3.5 ± 1.2 pmol/l; p < 0.001).

**Fig. 3:**
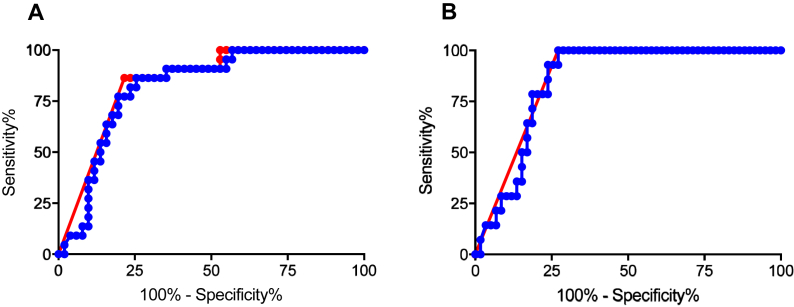
ROC curve analysis of AMH (red) and FSH (blue) at 24 months for (A) analysis of amenorrhoea versus ongoing menses (n = 22 and 51 respectively); (B) for diagnosis of POI versus not POI (n = 14 and 59 respectively). Data from all women in OPTION included.

The diagnostic accuracy of AMH and FSH for POI was assessed by ROC curve. There were no differences by goserelin treatment in women who were or were not amenorrhoeic at 12–24 months or who had POI at that time, thus for those analyses data from all women were used. For classification by amenorrhoea only, using hormone concentrations at 24 months, the ROC for AMH had an AUC of 0.84, sensitivity 86%, specificity 78%, LR 4.0 (Fig. 3A and table). Similarly, the ROC for FSH at 25 U/l had an AUC of 0.82, sensitivity 76%, specificity 71%, LR 2.1 (Fig. 3A). PPV, NPV and DOR calculations also showed a small advantage of AMH over FSH (Table 1).

ROC analysis for diagnosis of POI (Fig. 3B and Table 1) gave an AUC of 0.86 for AMH, sensitivity 100%, specificity 73%, and LR 3.7. For FSH, the AUC was 0.85 sensitivity 100%, specificity 66%, LR 3.0, again indicating the value of AMH for the diagnosis of POI, despite FSH>25 IU/l being one of the diagnostic criteria and hence sensitivity and NPV being 100%.

**Fig. 4:**
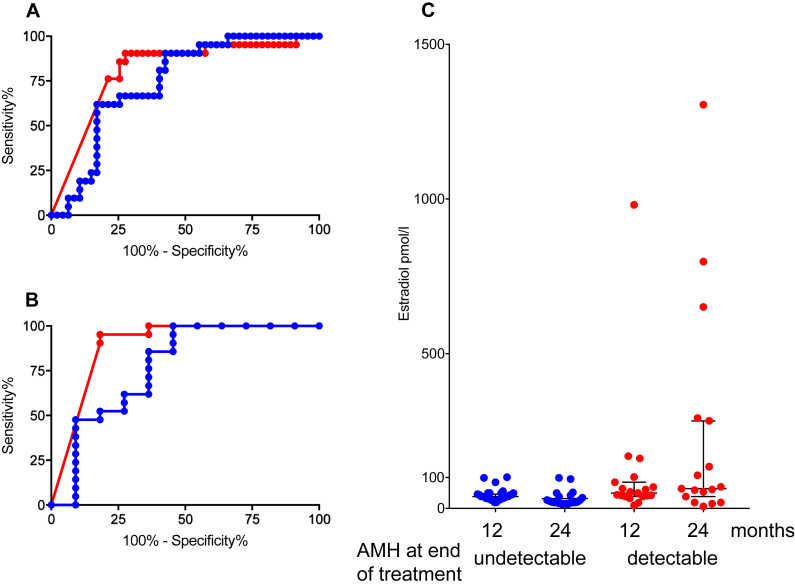
ROC curve analysis of (A) AMH (red) and FSH (blue) at end of treatment for prediction of POI versus not POI at 24 months (n = 23 and 45 respectively) for all women in the OPTION control group. (B) AMH (red) and FSH (blue) serum concentrations at end of treatment for prediction of POI versus not POI at 24 months for women aged >40 years in the control group in OPTION (n = 21 and 11 respectively). (C) Oestradiol concentrations at 12 and 24 months in women with undetectable AMH (blue) and detectable AMH (red) at the end of chemotherapy.

Data from the control group only were analysed to assess the value of AMH and FSH measured at the end of chemotherapy for prediction of POI at 24 months (Fig. 4A and table). The AUC for the AMH ROC was 0.84, sensitivity 78% specificity 82%, LR 4.4 and for FSH, the AUC was 0.72, sensitivity 91%, specificity 47%, LR 1.6. PPV and NPV analysis (table) also showed similar results, with a higher PPV for AMH despite the lower sensitivity, reflecting the poor specificity of FSH in this predictive analysis.

The importance of age in the recovery of ovarian function following chemotherapy was confirmed in this analysis (Fig. 2) with only two of 52 women aged ≤40 on whom AMH data were available developing POI. A ROC curve for the potential value of AMH measurement at the end of chemotherapy for prediction of POI was therefore calculated for women aged over 40 years at diagnosis in the control group, thus also avoiding any potential effect of goserelin (Fig. 4B and Table 2). The AUC for AMH was 0.89, sensitivity 91%, specificity 82% with LR 5.0, compared to AUC of 0.77, sensitivity 100%, specificity 55%, LR 2.2 for FSH. The PPV for AMH was 0.90, versus 0.81 for FSH. There were no E2 levels above 100 pmol/l at either later time point in women with undetectable AMH at end of treatment (Fig. 4C).

**Table 2:** Analysis of accuracy of AMH and FSH for diagnosis (at 24 months) and prediction (from end of chemotherapy [EOT] and pre-treatment versus POI at 24 months) of amenorrhoea and POI at 24 months.

Analysis	ROC AUC (95% CI)	Sensitivity	Specificity	LR	PPV	NPV	DOR
**Diagnosis by amenorrhoea at 24 months (n = 73)**							
AMH	0.84 (0.75–0.93)	0.86	0.78	4.0	0.63	0.93	23.0
FSH	0.81 (0.72–0.91)	0.86	0.71	2.9	0.56	0.92	15.2
**Diagnosis by POI at 24 months (n** = **73)**							
AMH	0.86 (0.78–0.95)	1.0	0.73	3.7	0.47	1.0	n/a
FSH	0.85 (0.77–0.94)	1.0	0.66	3.0	0.41	1.0	n/a
**Prediction of POI from EOT (all control group, n** = **68)**							
AMH	0.84 (0.73–0.94)	0.78	0.82	4.4	0.69	0.83	10.9
FSH	0.72 (0.60–0.84)	0.91	0.47	1.6	0.48	0.86	5.5
**Prediction of POI from EOT (age** > **40 control group only n** = **32)**							
AMH	0.89 (0.75–1.0)	0.91	0.82	5.0	0.90	0.82	42.8
FSH	0.77 (0.57–0.97)	1.0	0.55	2.2	0.81	1.0	n/a
**Prediction of POI by pre-treatment hormones (n** = **101)**							
AMH	0.77 (0.65–0.88)	0.95	0.49	9.3	0.30	0.98	17.1
FSH	0.72 (0.60–0.84)	0.89	0.43	7.7	0.27	0.95	6.5

For analysis of amenorrhoea and diagnosis of POI at 24 months, all women from OPTION are included where data are available. For prediction of POI, only women in the control group were included. For all ROC analysis, AUC p < 0.0001 except for prediction of POI from hormones at end of treatment in >40 age group: p = 0.0003 for AMH and p = 0.01 for FSH, and for pre-treatment prediction of POI, p = 0.0003 for AMH, p = 0.028 for FSH.

The predictive value of pre-treatment hormone concentrations was also assessed, categorising women as POI or not, using data from all women (n = 101; Table 2). For AMH the AUC was 0.77, with peak LR 9.3 at AMH of <7.3 pmol/l, sensitivity 95%, specificity 49%, PPV 0.30 and NPV 0.98. For FSH, the AUC was 0.72, with peak LR 7.7 at FSH >4.3 IU/l, at which concentration sensitivity was 89% and specificity 43%, PPV 0.27, NPV 0.95.

## 4 Discussion

These data indicate that measurement of AMH following chemotherapy for breast cancer using the improved sensitivity of the Roche Diagnostics automated Elecsys^®^ assay is an accurate diagnostic test of menopausal status after recovery from treatment, and that analysis at the end of chemotherapy may predict POI. For the predictive analysis, ROC curve analysis gave an AUC of 0.89, likelihood ratio of 5.0 and DOR of 42.8: values for AUC > 0.9, LR > 7 and DOR > 20 are regarded as indicating high accuracy [25] supporting the potential value of this biomarker. Analysis of AMH as a diagnostic test for POI at 24 months after diagnosis indicated substantially greater accuracy than in a previous similar analysis in women treated for breast cancer when AMH was measured using a less sensitive assay [26].

The menstrual and endocrine changes of the menopausal transition have been documented in detail in normal women, but that classification specifically excludes women treated with chemotherapy [27]. The choice of endocrine agent after chemotherapy depends on menopausal status. Prediction of POI is well established to be dependent on age [6], [7], [13] but the value of biochemical markers has been unclear [28]. Thus, in a recent analysis, women showing ovarian recovery did not show differential FSH concentrations [29]. We have previously suggested that high-sensitivity AMH assays may be of value in this situation [22]. Here we have performed a more detailed analysis using a larger independent cohort of women, and assessed the value of post-chemotherapy AMH as a predictor of later POI. Despite including a threshold level of FSH in the classification of POI, AMH performed better than FSH.

Analysis of AMH and FSH as diagnostic tests for amenorrhoea *versus* menses at 24 months showed that an undetectable AMH level gave high sensitivity and specificity with ROC values for AUC, with sensitivity and specificity all better than for FSH. Classification of women as having POI or not also showed high diagnostic accuracy with AMH (sensitivity 100%; specificity 73%), thus AMH was undetectable using this highly sensitive assay in all women with POI at 24 months. Tamoxifen did not affect AMH concentrations, as previously reported [30], [31].

The potential value of AMH at the end of chemotherapy in identifying POI was examined. AMH levels were very low in all women following chemotherapy, but were markedly higher in women who did not subsequently have POI at 24 months compared to those who did, highlighting the value of using this assay with improved sensitivity. Thereafter a small increase in AMH was seen in those women who were later classified as not having POI, whereas there was no recovery in those who with POI. This shows the value of AMH in identifying even very low levels of ovarian follicular activity in this context.

Because of the impact of goserelin treatment on hormone concentrations at the end of chemotherapy, further analyses of the predictive value of AMH and FSH were performed using data only from the control group, and from women aged over 40 as the initial analysis confirmed the importance of age as a predictor of recovery [7], [10], [13]. AMH also showed significant value in the prediction of recovery of ovarian function with FSH showing lower diagnostic accuracy. The higher specificity for AMH demonstrates its ability to reflect very low levels of ovarian activity when FSH is in the menopausal range, activity that will then increase in many women after chemotherapy. AMH may also be only transiently detectable at the end of chemotherapy in some women, indicating menopausal onset after ageing-related loss of a very small amount of residual activity present at the end of chemotherapy.

A small proportion of women, even those in their late 40s at diagnosis, can show late recovery of ovarian function [32]. In this analysis, POI at 24 months was used, thus few women would be likely to have amenorrhoea/POI at that time and still show later recovery of ovarian function, but any such women would reduce the diagnostic accuracies reported here. A further limitation of this study is the need to include only the control group (i.e. not treated with goserelin) in the predictive analysis, thus reducing the number of women evaluable.

Pre-treatment AMH was confirmed to be predictive of POI following chemotherapy [9], [10], [33]. An optimal cut-point of 7.3 pmol/l pre-treatment was identified, which showed good sensitivity but poor specificity. While age is also an important predictor [6], [10], this information may be of value in identifying women at particular risk of loss of fertility following chemotherapy, although fertility can be retained in women with even very low AMH levels [34]. Conversely, AMH above that threshold showed very good prediction of not having POI at 24 months.

In conclusion, these data show that using a highly sensitive assay, measurement of AMH at the end of chemotherapy can identify women who will show ovarian recovery with good precision. Conversely, undetectable AMH at end of chemotherapy may be useful in identifying women who will not show ovarian recovery, which could influence choice of endocrine therapy by avoiding the need for ovarian suppression. Larger prospective trials are needed to validate the role of AMH in oncology clinical practice.

## Conflict of interest statement

RAA has undertaken consultancy work for Roche and Boehringer Ingelheim. The other authors have no conflicts of interest to declare.

## Grant support

The OPTION trial was funded by Cancer Research UK (ref C4831/A4181). We are grateful to Roche Diagnostics for the provision of hormone assay materials.This work was undertaken in the MRC Centre for Reproductive Health which is funded by the MRC Centre grant MR/N022556/1

## References

[1] Early Breast Cancer Trialists' Collaborative G, Dowsett M., Forbes J.F., Bradley R., Ingle J., Aihara T. (2015). Aromatase inhibitors versus tamoxifen in early breast cancer: patient-level meta-analysis of the randomised trials. Lancet.

[2] Dowsett M., Stein R.C., Coombes R.C. (1992). Aromatization inhibition alone or in combination with GnRH agonists for the treatment of premenopausal breast cancer patients. J Steroid Biochem Mol Biol.

[3] Pagani O., Regan M.M., Walley B.A., Fleming G.F., Colleoni M., Lang I. (2014). Adjuvant exemestane with ovarian suppression in premenopausal breast cancer. N Engl J Med.

[4] Francis P.A., Regan M.M., Fleming G.F., Lang I., Ciruelos E., Bellet M. (2015). Adjuvant ovarian suppression in premenopausal breast cancer. N Engl J Med.

[5] Bernhard J., Luo W., Ribi K., Colleoni M., Burstein H.J., Tondini C. (2015). Patient-reported outcomes with adjuvant exemestane versus tamoxifen in premenopausal women with early breast cancer undergoing ovarian suppression (TEXT and SOFT): a combined analysis of two phase 3 randomised trials. Lancet Oncol.

[6] Petrek J.A., Naughton M.J., Case L.D., Paskett E.D., Naftalis E.Z., Singletary S.E. (2006). Incidence, time course, and determinants of menstrual bleeding after breast cancer treatment: a prospective study. J Clin Oncol.

[7] Jacobson M.H., Mertens A.C., Spencer J.B., Manatunga A.K., Howards P.P. (2016). Menses resumption after cancer treatment-induced amenorrhea occurs early or not at all. Fertil Steril.

[8] Smith I.E., Dowsett M., Yap Y.S., Walsh G., Lonning P.E., Santen R.J. (2006). Adjuvant aromatase inhibitors for early breast cancer after chemotherapy-induced amenorrhoea: caution and suggested guidelines. J Clin Oncol.

[9] Anderson R.A., Cameron D.A. (2011). Pretreatment serum anti-mullerian hormone predicts long-term ovarian function and bone mass after chemotherapy for early breast cancer. J Clin Endocrinol Metab.

[10] Su H.C., Haunschild C., Chung K., Komrokian S., Boles S., Sammel M.D. (2014). Prechemotherapy antimullerian hormone, age, and body size predict timing of return of ovarian function in young breast cancer patients. Cancer.

[11] Ruddy K.J., O'Neill A., Miller K.D., Schneider B.P., Baker E., Sparano J.A. (2014). Biomarker prediction of chemotherapy-related amenorrhea in premenopausal women with breast cancer participating in E5103. Breast Cancer Res Treat.

[12] Gracia C.R., Sammel M.D., Freeman E., Prewitt M., Carlson C., Ray A. (2012). Impact of cancer therapies on ovarian reserve. Fertil Steril.

[13] Vriens I.J., De Bie A.J., Aarts M.J., de Boer M., van Hellemond I.E., Roijen J.H. (2017). The correlation of age with chemotherapy-induced ovarian function failure in breast cancer patients. Oncotarget.

[14] Dewailly D., Andersen C.Y., Balen A., Broekmans F., Dilaver N., Fanchin R. (2014). The physiology and clinical utility of anti-Mullerian hormone in women. Hum Reprod Update.

[15] Anderson R.A., Themmen A.P.N., Al Qahtani A., Groome N.P., Cameron D.A. (2006). The effects of chemotherapy and long-term gonadotrophin suppression on the ovarian reserve in premenopausal women with breast cancer. Hum Reprod.

[16] Decanter C., Morschhauser F., Pigny P., Lefebvre C., Gallo C., Dewailly D. (2010). Anti-Mullerian hormone follow-up in young women treated by chemotherapy for lymphoma: preliminary results. Reprod Biomed Online.

[17] Partridge A.H., Ruddy K.J., Gelber S., Schapira L., Abusief M., Meyer M. (2010). Ovarian reserve in women who remain premenopausal after chemotherapy for early stage breast cancer. Fertil Steril.

[18] Su H.I., Sammel M.D., Green J., Velders L., Stankiewicz C., Matro J. (2010). Antimullerian hormone and inhibin B are hormone measures of ovarian function in late reproductive-aged breast cancer survivors. Cancer.

[19] Dillon K.E., Sammel M.D., Prewitt M., Ginsberg J.P., Walker D., Mersereau J.E. (2013). Pretreatment antimullerian hormone levels determine rate of posttherapy ovarian reserve recovery: acute changes in ovarian reserve during and after chemotherapy. Fertil Steril.

[20] Sowers M.R., Eyvazzadeh A.D., McConnell D., Yosef M., Jannausch M.L., Zhang D. (2008). Anti-Mullerian hormone and inhibin B in the definition of ovarian aging and the menopause transition. J Clin Endocrinol Metab.

[21] Anderson R.A., Anckaert E., Bosch E., Dewailly D., Dunlop C.E., Fehr D. (2015). Prospective study into the value of the automated Elecsys antimullerian hormone assay for the assessment of the ovarian growing follicle pool. Fertil Steril.

[22] Chai J., Howie A.F., Cameron D.A., Anderson R.A. (2014). A highly-sensitive anti-Mullerian hormone assay improves analysis of ovarian function following chemotherapy for early breast cancer. Eur J Cancer.

[23] Leonard R., Adamson D., Bertelli G., Mansi J., Yellowlees A., Dunlop J. (2017). GnRH agonist for protection against ovarian toxicity during chemotherapy for early breast cancer: the Anglo Celtic Group OPTION trial. Ann Oncol.

[24] ESHRE Guideline Group on POI, Webber L., Davies M., Anderson R., Bartlett J., Braat D. (2016). ESHRE Guideline: management of women with premature ovarian insufficiency. Hum Reprod.

[25] Fischer J.E., Bachmann L.M., Jaeschke R. (2003). A readers' guide to the interpretation of diagnostic test properties: clinical example of sepsis. Intensive Care Med.

[26] Su H.I., Chung K., Sammel M.D., Gracia C.R., DeMichele A. (2011). Antral follicle count provides additive information to hormone measures for determining ovarian function in breast cancer survivors. Fertil Steril.

[27] Harlow S.D., Gass M., Hall J.E., Lobo R., Maki P., Rebar R.W. (2012). Executive summary of the Stages of Reproductive Aging Workshop + 10: addressing the unfinished agenda of staging reproductive aging. J Clin Endocrinol Metab.

[28] Torino F., Barnabei A., De Vecchis L., Sini V., Schittulli F., Marchetti P. (2014). Chemotherapy-induced ovarian toxicity in patients affected by endocrine-responsive early breast cancer. Crit Rev Oncol Hematol.

[29] Krekow L.K., Hellerstedt B.A., Collea R.P., Papish S., Diggikar S.M., Resta R. (2016). Incidence and predictive factors for recovery of ovarian function in amenorrheic women in their 40s treated with letrozole. J Clin Oncol.

[30] Anderson R.A., Cameron D.A. (2007). Assessment of the effect of chemotherapy on ovarian function in women with breast cancer. J Clin Oncol.

[31] Dezellus A., Barriere P., Campone M., Lemanski C., Vanlemmens L., Mignot L. (2017). Prospective evaluation of serum anti-Mullerian hormone dynamics in 250 women of reproductive age treated with chemotherapy for breast cancer. Eur J Cancer.

[32] van Hellemond I.E.G., Vriens I.J.H., Peer P.G.M., Swinkels A.C.P., Smorenburg C.H., Seynaeve C.M. (2017). Ovarian function recovery during anastrozole in breast cancer patients with chemotherapy-induced ovarian function failure. JNCI JNatl Cancer Inst.

[33] Anderson R.A., Rosendahl M., Kelsey T.W., Cameron D.A. (2013). Pretreatment anti-Mullerian hormone predicts for loss of ovarian function after chemotherapy for early breast cancer. Eur J Cancer.

[34] Hamy A.S., Porcher R., Eskenazi S., Cuvier C., Giacchetti S., Coussy F. (2016). Anti-Mullerian hormone in breast cancer patients treated with chemotherapy: a retrospective evaluation of subsequent pregnancies. Reprod Biomed Online.

